# Antibody-Based Array for Tacrolimus Immunosuppressant Monitoring with Planar Plastic Waveguides Activated with an Aminodextran-Lipase Conjugate

**DOI:** 10.1021/acs.analchem.4c02028

**Published:** 2024-08-22

**Authors:** Bettina Glahn-Martínez, Sonia Herranz, Elena Benito-Peña, Guillermo Orellana, Maria C. Moreno-Bondi

**Affiliations:** † Department of Analytical Chemistry, Faculty of Chemistry, Universidad Complutense de Madrid, Plaza de las Ciencias 2, Madrid 28040, Spain; ‡ Department of Organic Chemistry, Faculty of Chemistry, Universidad Complutense de Madrid, Plaza de las Ciencias 2, Madrid 28040, Spain

## Abstract

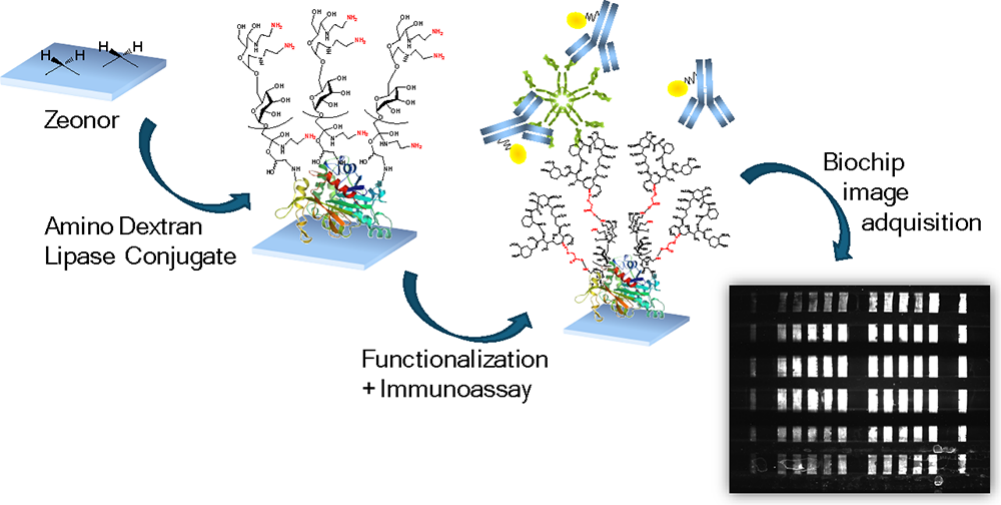

Cyclic olefin copolymers (COC; e.g., Zeonor, Topas, Arton, etc.) are materials with outstanding properties for developing point-of-care systems; however, the lack of functional groups in their native form makes their application challenging. This work evaluates different strategies to functionalize commercially available Zeonor substrates, including oxygen plasma treatment, photochemical grafting, and direct surface amination using an amino dextran-lipase conjugate (ADLC). The modified surfaces were characterized by contact angle measurements, Fourier transform infrared-attenuated total reflection analysis, and fluorescence assays based on evanescent wave excitation. The bioaffinity activation through the ADLC approach results in a fast, simple, and reproducible approach that can be used further to conjugate carboxylated small molecules (e.g., haptens). The usefulness of this approach has been demonstrated by the development of a heterogeneous fluorescence immunoassay to detect tacrolimus (FK506) immunosuppressant drug using an array biosensor platform based on evanescence wave laser excitation and Zeonor-ADLC substrates. Surface modification with ADLC-bearing FK506 provides a 3D layer that efficiently leads to a remarkably low limit of detection (0.02 ng/mL) and IC50 (0.9 ng/mL) together with a wide dynamic range (0.07–11.3 ng/mL).

Tacrolimus (FK506) is a hydrophobic macrolide lactone produced by *Streptomyces tsukubaensis* and is widely used as an immunosuppressant drug after organ transplantation.^1^ The free fraction of this drug in serum (1–2%) correlates with its accumulation in blood cells.^2^ Due to its narrow therapeutic window, in situ (semi)continuous monitoring of FK506 in transplanted patients would be most helpful in boosting its therapeutic efficiency and avoiding potentially adverse effects. In this context, microdialysis systems interfaced with optical biosensors^3^ could achieve the required quantification of FK506 and its metabolites in plasma thanks to their intrinsic high sensitivity, selectivity, and multiplexing capability (sensor arrays).

Point-of-care (POC) microdevices, including miniaturized platforms such as microfluidic devices and biosensors, offer all these features, particularly those fabricated in plastic materials such as cyclic olefin copolymers (COC).^3^ In recent years, COC polymers have drawn attention as chip substrates for biomedical diagnostics due to their suitable optical properties (good transmittance in the near-UV region, low autofluorescence), much lower protein binding than glass and other plastic substrates,^4,5^ negligible water uptake, low permeability for water vapor, and ease of fabrication.^3,6^ Furthermore, they are compatible with bioreagents and biological samples, withstanding polar organic solvents typically used in life sciences (e.g., isopropanol, acetone).^6,7^ Notably, some types of COCs, namely Zeonor and Zeonex, have been used for DNA immobilization, nucleic acid purification, and microarray fabrication.^8,9^

However, these materials lack functional groups due to their pure hydrocarbon composition, making surface modification difficult. The hydrophobic nature of the COCs can promote fouling by proteins, which is undesirable in applications for which protein adhesion is not required.

Various surface activation techniques have been proposed to overcome those challenges, including oxygen plasma treatment, UV ozonolysis, or photografting.^9−11^ These methods aim to introduce hydrophilic polar functional groups to increase the surface free energy and minimize fouling by proteins. However, during the chemical modification process, the polymer surface may undergo oxidation, degradation, and cross-linking, which can cause structural alterations to its first few molecular layers,^12^ thereby affecting the ability of light to propagate through COC substrates and preventing the generation of its evanescent field.^13^ Additionally, batch-to-batch reproducibility is conditioned by the instability of these activated surfaces because polymer chains on the surface tend to rearrange stochastically and partially return to the native hydrophobic form.^14^ Currently, a large number of POC devices employ waveguides to achieve measurements based on total internal reflection;^15^ therefore, it is essential to maintain a correct propagation of the light through the substrate. For these reasons, an extensive effort has been devoted to implement new biomolecule immobilization strategies on COC substrates to improve uniformity, tethering stability, and density of active ligands.^6,12,16^

Lipases are enzymes that contain both hydrophilic and hydrophobic regions on their surface, which allows them to adsorb onto hydrophobic surfaces through a mechanism known as interfacial adsorption.^17^ Such enzymes also feature remarkable stability; for instance, BTL2 produced by the thermophilic bacterium *Geobacillus thermocatenulatus* shows high thermal stability at intermediate temperatures (50 °C) and in alkaline media or organic solvents.^18,19^ These properties suggest that lipases might be useful tools for streamlined COC functionalization and serve as scaffolds to further immobilize a variety of molecules, such as haptens or antibodies.

Chemically modified lipases can also be used for surface functionalization. For example, we have previously applied lipases modified with a dextran polysaccharide to develop microarray biosensors. The two-faced Janus material provided a flexible hydrophilic network to immobilize the hepatoxic microcystin LR (MCLR) in a high conjugation degree. The MCLR-dextran-lipase chimeras were immobilized by nonspecific hydrophobic interactions on planar glass substrates, thus improving the interaction with corresponding antibodies and leading to increased sensitivity and a larger dynamic range of the bioassay.^20,21^

In this work, the surface functionalization of commercially available Zeonor substrates was investigated using three strategies: oxygen plasma treatment, photochemical grafting, and direct surface amination using an aminated dextran-BTL2 conjugate (ADLC). Surface modification procedures were characterized by contact angle measurements, Fourier transform infrared-attenuated total reflection spectroscopy (FTIR-ATR), and atomic force microscopy. The suitability of the different approaches for the modification of the COC substrate has been assessed through a heterogeneous competitive fluorescence immunoassay to detect tacrolimus (FK506) (Figure 1).

**Figure 1:**
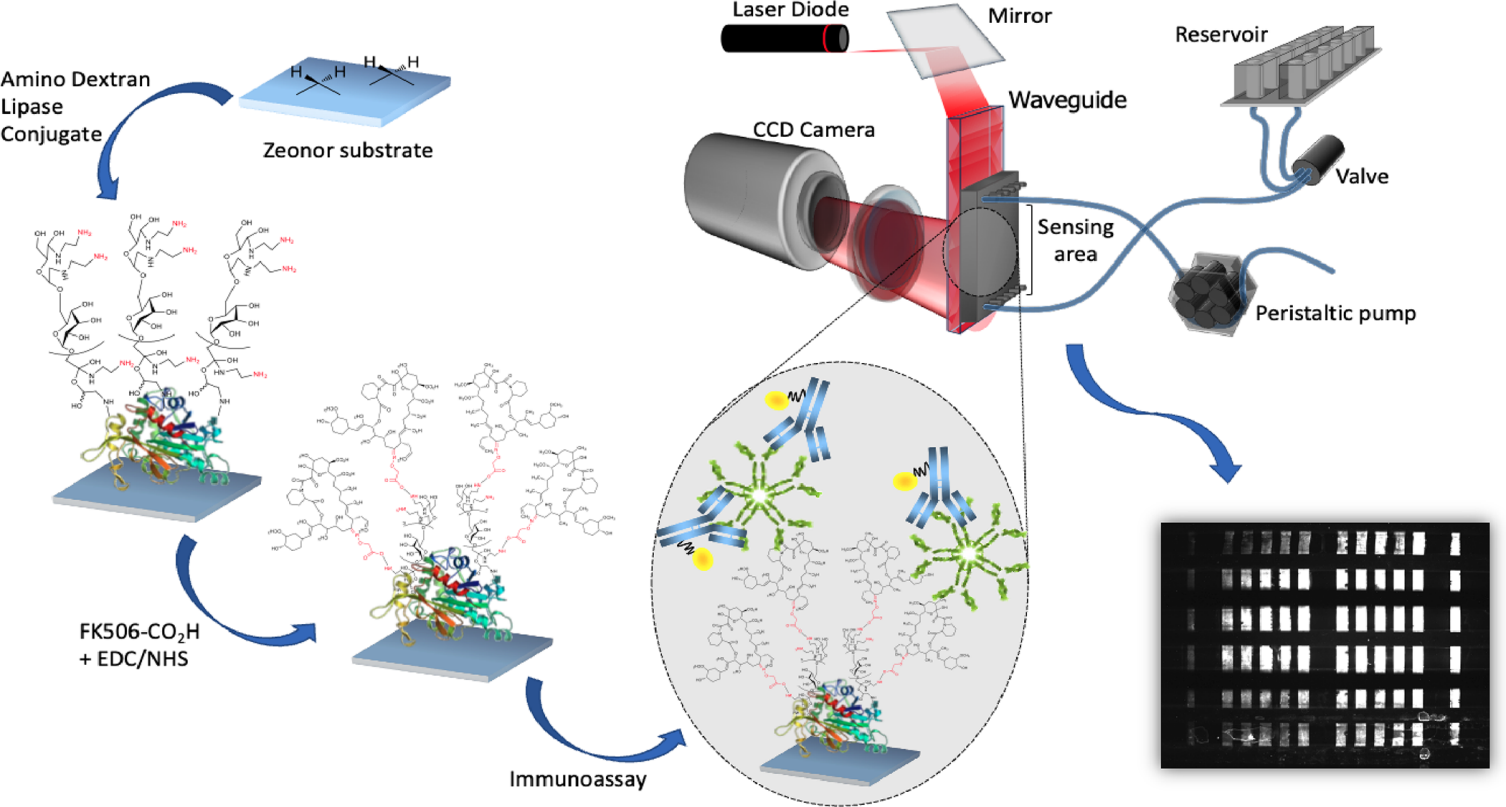
Workflow of the developed competitive fluorescence immunoassay for the detection of FK506. (Left) Activation of the COC waveguides through the amino dextran-lipase conjugate and subsequent covalent binding of FK506-CO_2_H. (Right) Measurement and image acquisition: The array was then incubated with the sample containing a mixture of a positive control and anti-FK506 antibodies, followed by an incubation with the labeled detection antibodies.

## Methods

### Surface Amination Using an Amino Dextran-Lipase Conjugate

In this novel approach, amino groups are introduced into specific areas of the Zeonor waveguide using a modified BTL2 lipase, which is adsorbed efficiently on the highly hydrophobic polymer surfaces. As previously described,^20^ aminated dextran-BTL2 conjugates were hydrophobically adsorbed using a 15-channel PDMS patterning gasket placed over the functionalized waveguide. A 0.05 mg/mL conjugate solution in PBS (pH 7.4) was injected into each channel. After 30 min of incubation at room temperature, the channels were washed with 0.5 mL of PBS. The reaction scheme is depicted in Figure S1c.

### Antigen Immobilization onto the Zeonor-Activated Sensing Surface

The functionalization of Zeonor substrates activated with primary amino groups, with the carboxylated FK506 hapten (FK506-CO_2_H) and the positive control (biotin), was carried out through their acidic groups by the active ester method.^22^

The active ester method was developed in one step in specific waveguide areas. For this purpose, a PDMS mold was arranged on the surface of aminated Zeonor, forming 15 channels parallel to each other. Each channel was filled with about 50 μL of a solution containing FK506-CO_2_H (100 μg/mL) or biotin (60 μg/mL), EDC (100 mM) and NHS (50 mM) in MES buffer (50 mM, pH 6.0), using hypodermic needles and disposable syringes. The mixtures were incubated on Zeonor substrates for at least 4 h at 4 °C and protected from light. Subsequently, the mold was removed from the surface and rinsed with deionized water. The patterned slides were blocked in PBSPF for 1 h, rinsed with deionized water, dried under argon, and either introduced into the Leopard Array for the measurements or stored at 4 °C.

### Assay Protocol

The working principle of the assay is based on a competitive inhibition between FK506-CO_2_H, immobilized onto the Zeonor surface, and the FK506 in the sample for the antibody binding sites. The assay occurs only in the overlapping regions between those functionalized with the hapten and those of the assay channels, which means 90 sensing regions, 15 for each sample.

As described previously, the patterned modified Zeonor slides were placed in the array biosensor instrument. FK506 standard solutions (390 μL, 0.0001–100 μg/mL) were mixed with 10 μL of Ab solution (0.5 μg/mL IgG anti-FK506 and 0.5 μg/mL IgG antibiotin). After 5 min of preincubation at room temperature, the mixture was loaded into the sample reservoirs, pumped through the channels, and incubated statically over the sensor surface for 20 min. Then, the channels were rinsed twice with 800 μL of buffer, and 400 μL of a solution containing 2.5 μg/mL of labeled Abs in PBS was used to reveal the surface pattern of primary antibodies bound to the patterned antigens. This solution was pumped to the sensor surface through the channels and incubated for 20 min statically. The unbound labeled Abs was removed by rinsing four times the channels with 800 μL of PBS, and the slide was then imaged. A scheme of the immunoassay workflow is depicted in Figure S2a.

After the immunoaffinity reaction, the radiation from the diode laser uniformly illuminates the Zeonor substrate, and the produced evanescent wave excites the fluorescent molecules located in the sensing area. Intensity data were extracted from the CCD images (15 s, 21.5 gain units) and normalized as described elsewhere.^23^ The positions and intensities of the fluorescent regions on the Zeonor surface allow for identification and quantification.

The fluorescence data were collected as the *B* (fluorescence signal in the presence of FK506), *B*_0_ (fluorescence signal in the absence of the analyte), and the response normalized using the following expression:
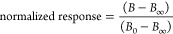
1where *B*_∞_ is the background fluorescence obtained in the presence of an excess of FK506. The normalized experimental data were plotted as a function of the FK506 concentration in logarithmic scale and fitted to a four-parameter sigmoidal logistic equation (eq 2) using the Origin 2019 software:
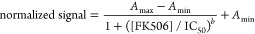
2where *A*_max_ and *A*_min_ correspond, respectively, to the asymptotic maximum and minimum of the normalized signal, IC_50_ is the concentration of analyte at the inflection point (concentration that provides 50% inhibition of *A*_max_), and *b* represents the slope of the curve at the inflection point. The limit of detection (LOD) corresponds to the analyte concentration for which the tracer binding to the antibody was inhibited by 10%, and the dynamic range (DR) of the method was calculated as the analyte concentrations that produced a normalized signal in the 20–80% range defined by the *A*_max_ and *A*_min_ asymptotes. The fluorescence signals were normalized with positive controls (biotin) to minimize variability between channels and slides.^23^

## Results and Discussion

### Surface Treatment

As it was mentioned above, this work addresses different strategies to the challenging functionalization of the surface of the COCs, to achieve the most efficient immobilization of FK506-CO_2_H.

#### Feasibility of the Oxygen Plasma Treatment

Plasma treatment was carried out at two typical etching times (3 and 6 min), and the oxidation achieved through this protocol was assessed by measuring the degree of hydrophilicity by the water contact angle. Figure S3 shows the contact angles on Zeonor substrates after 0, 3, and 6 min of oxygen plasma treatment. The untreated Zeonor substrate exhibits a large contact angle (>75°) due to its highly hydrophobic nature. Interestingly, an exposure of at least 3 min to the oxygen plasma resulted in a marked decrease of the contact angles (<20°), achieving almost complete wettability in both cases and comparable to a conventional glass waveguide substrate (Figure S3).

The oxidized substrates were treated with 3-aminopropyltriethoxysilane (APTES, 3%, v/v) to introduce amino groups that react with FK506-CO_2_H and allow its covalent attachment to the substrate through an amide bond.^24^ The behavior of the surface after functionalization with different concentrations of FK506-CO_2_H (90–900 μg/mL) showed a weak response when using the substrates exposed to plasma for 3 min (Figure S4a), and no response was obtained in the case of Zeonor treated for 6 min (Figure S4b).

The relatively low sensitivity obtained under these conditions might be attributed to the deterioration of the Zeonor optical properties upon the plasma treatment.^25^ To confirm this adverse effect, the efficiency of the evanescent waves propagated through the Zeonor waveguide was evaluated by exposing a 0.5 μg/mL aqueous solution of AlexaFluor 647-anti-IgG to the evanescent field generated by total internal reflection (TIR) laser illumination at 635 nm. Fluorescence signals were extracted from the CCD images as pixel intensity values. As shown in Figure S5, the loss of excitation efficiency using Zeonor substrates exposed to 3 or 6 min of oxygen plasma was close to 60% with respect to the untreated surface. These results are comparable to those described in the literature for similar cyclo-olefin polymers (e.g., Zeonex, Topas), where extensive loss of light transmission was observed over almost the entire visible wavelength range, probably due to the formation of light-absorbing species and photo-oxidation reactions.^25,26^

Additionally, this analysis lends confirmation to the excellent optical characteristics of the original Zeonor, displaying a 130% better signal-to-noise (S/N) response compared to glass substrates.

#### Feasibility of the Photochemical Modification

Among the various surface modification methods, photoinduced graft polymerization has been proven to be suitable for introducing acrylic and methacrylic monomers on COC surfaces.^11,27^ These processes are based on the use of hydrogen abstraction photoinitiators such as benzophenone, anthraquinones, or the like.^28,29^ Therefore, this technique was also evaluated for the introduction of ω-amino groups on the Zeonor surfaces. The latter was achieved in two steps: (i) generation of surface-bonded initiators when UV-excited benzophenone (BP) or azobis(isobutyronitrile) (AIBN) abstract hydrogen atoms from the polymer surface; (ii) grafted polymerization for 30 min of 2-aminoethyl methacrylate (2-AEM) under UV-irradiation (312 nm) using a constant monomer concentration.

The effect of grafting was monitored by ATR-FTIR. Figure S6a shows the ATR-FTIR spectra corresponding to original and different 2-AEM-grafted Zeonor substrates (deoxygenated 0.1 M solution in 90:10 v/v MeOH-water) and increasing concentrations of the benzophenone initiator (0.5–5.0%, w/v). The ATR-FTIR spectra display the typical absorption bands of polyolefins,^30^ including the symmetric and asymmetric vibrations of the C–H and C–C bonds at 2917 and 2845 cm^–1^, respectively, and the peaks around 1470 cm^–1^ corresponding to the deformation of the methylene group. However, the main absorption bands of the amino group (two bands at ca. 3400 cm^–1^ corresponding to the N–H stretch vibration) and the strong band of the C=O group (at ca. 1740 cm^–1^) were not observed, indicating the very low functionalization efficiency of the photochemical method.

To confirm these observations, conventional fluorescence-linked immunoassay (FLI) was performed using the aminated Zeonor substrates and following the procedure described above. As shown in Figure S6b, no response was observed in any of the cases. Similar results were obtained with AIBN as the photoinitiator; therefore, this functionalization strategy was discarded for further experiments.

#### Feasibility of the Aminated Dextran-Lipase Modification

The lipase selected to carry out the synthesis of the conjugates prepared in this work was BTL2 from *Geobacillus thermocatenulatus*, because it shows a good thermal stability in alkaline media or in the presence of organic solvents, allowing its chemical modification and conjugation to various molecules and polymers. Lipase was derivatized with dextran in the solid phase, following the steps previously described.^20^ The efficient BTL2 modification with the polysaccharide was confirmed by Raman and ATR-FTIR (Figure S7) spectroscopies, and the amount of amino groups in the ADLC conjugates (ca. 0.1 μmol/mg) was assessed by the trinitrobenzenesulfonic acid (TNBS) test.^20,21^ Also, the enzyme activity was evaluated after the chemical modification and was not significantly affected, retaining more than 80% of the initial enzymatic activity.

The Zeonor amination was performed in just one step by immobilizing the ADLC conjugate onto the planar waveguide through intermolecular hydrophobic interactions. The BTL2 lipase interacts with any nonpolar surface like they do on fat surfaces, and this interaction is quite specific since it adsorbs on hydrophobic surfaces at low ionic strength, the condition under which most proteins cannot do it. The lipase-hydrophobic surface complex is significantly stable and can be used in aqueous and anhydrous media.^18,19^ We have already demonstrated the suitability of ADCL conjugates as tools for low molecular weight ligand immobilization in FLI development.^20^ Now, we introduce a novel strategy in which the immobilization process is greatly simplified by using substrates of a hydrophobic nature, avoiding the tedious process of bringing hydrophobicity to surfaces as polar as glass waveguides.

**Figure 2:**
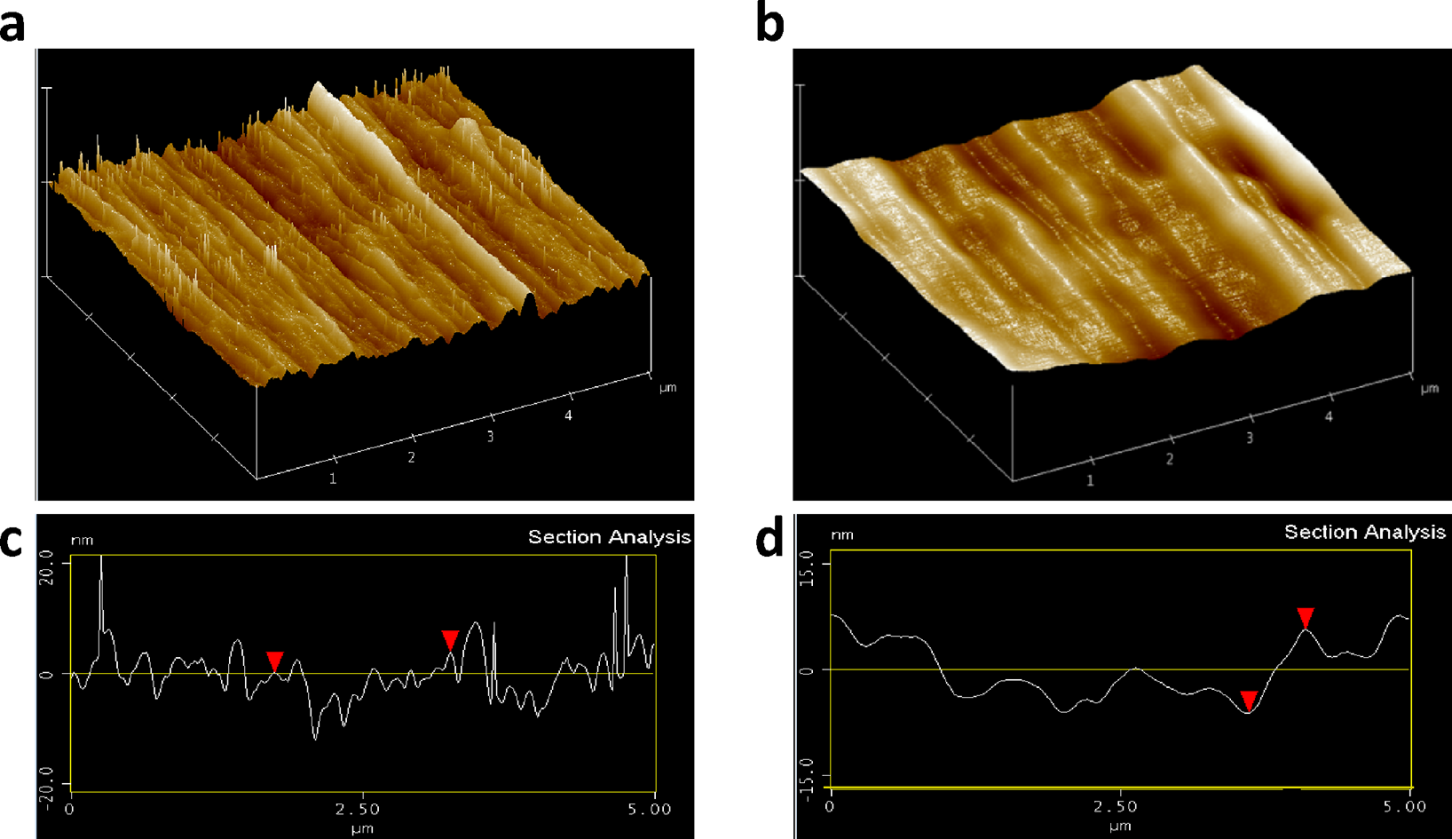
AFM topography of the planar Zeonor surface used as a waveguide: (a) original Zeonor substrate; (b) Zeonor functionalized with the ADLC solution (50 μg/mL, 30 min). (c, d) Topographic profiles along panels (a) and (b), respectively.

To evaluate the adsorption of the ADLC conjugate on Zeonor surfaces, its topography was studied by AFM. Figure 2a–d shows the images obtained for a planar Zeonor waveguide before and after immobilization of the ADLC (50 μg/mL, 30 min incubation). As can be observed therein, the AFM images evidence the effective immobilization of the ADLC on the Zeonor surface since smoothing of the surface topography is noticed (decrease of the average vertical distance of ca. 11 nm). This observation is compatible with previous results^20^ that suggest the formation of a monolayer of enzyme onto the Zeonor surface.

The immobilization procedure was optimized by studying several parameters that affect the efficiency of the FK506-CO_2_H immobilization on the ADLC-coated Zeonor, namely, ADLC concentration, nature and pH of the incubating solution, 1-ethyl-3-(3-(dimethylamino)propyl) carbodiimide (EDC) concentration, and the viability of using *N*-hydroxysuccinimide (NHS).

### Surface Functionalization with ADLC

**Figure 3:**
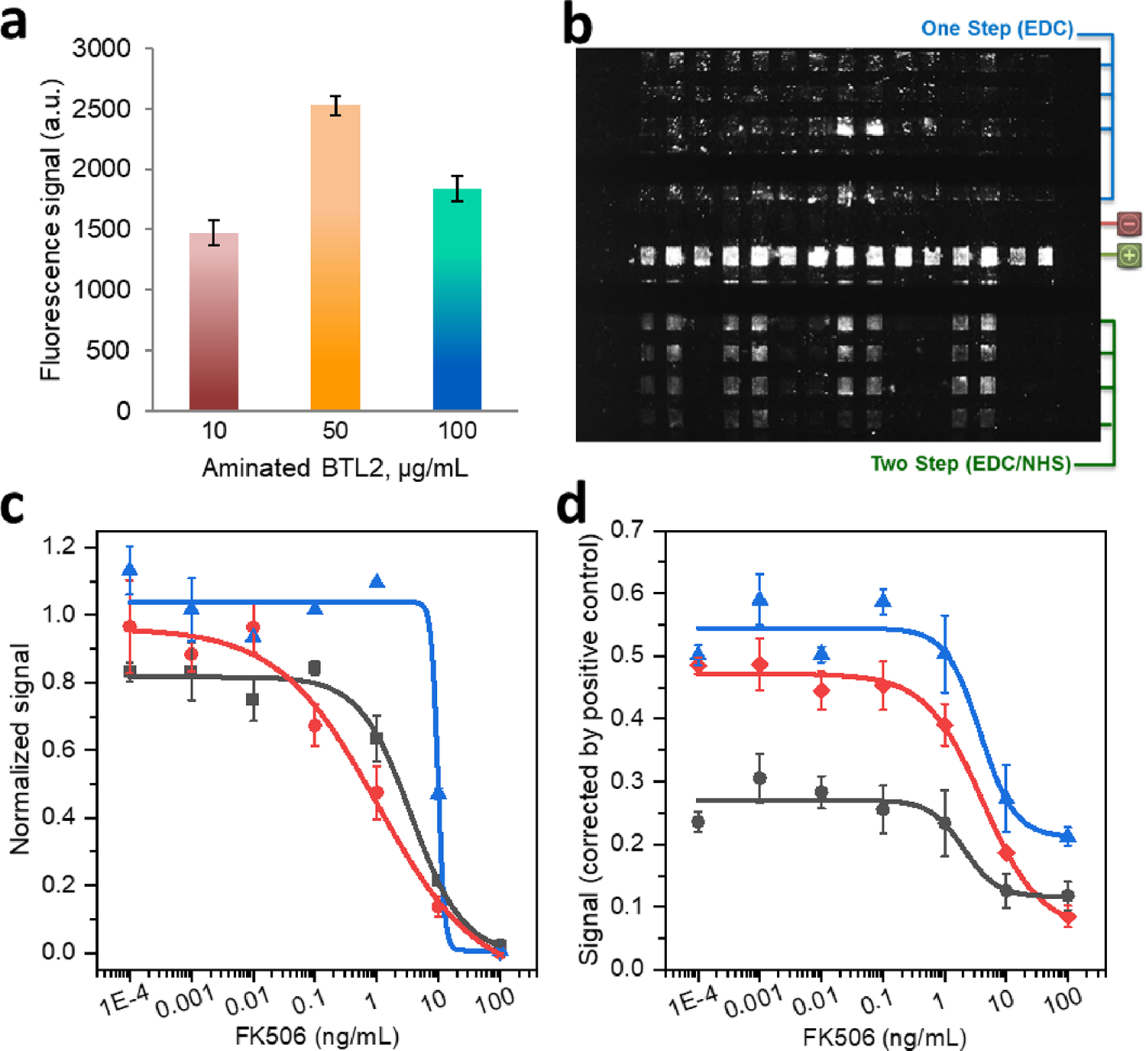
(a) Comparison of the efficiency of different amino dextran-BTL2 coating solutions at 10, 50, and 100 μg/mL. The conjugate-activated slide was patterned with FK506-CO_2_H (100 μg/mL) and incubated with 0.5 μg/mL anti-FK506 in the absence of free analyte. A 2.5 μg/mL Alexa Fluor 647-anti-IgM solution was used as the developer agent. (b) Biochip image obtained using two different methods for covalent coupling of FK506-CO_2_H: the one-step method (EDC) (blue) and the two-step method (EDC/NHS) (green). (c) Competitive calibration curve obtained at 62.5 (black square), 100 (red circle), and 200 μM (blue triangle) of EDC, using a fixed concentration of 50 mM NHS and 100 μg/mL FK506-CO_2_H. (d) Competitive calibration curve obtained at 50 (black circle), 100 (red diamond), and 200 μg/mL (blue triangle) of FK506-CO_2_H, with fixed concentrations of 50 mM NHS and 62.5 μM EDC. The assays were performed with FK506 (0.0001–100 μg/L) in the presence of 0.5 μg/mL anti-FK506 and antibiotin antibodies. A mixture of 2.5 μg/mL Alexa Fluor647-anti-IgM and Alexa Fluor 647-anti-IgG was used as the tracer solution. The results are mean signals ± standard errors of the mean (*n* ≥ 3).

The sensor was fabricated by immobilizing the ADLC conjugate onto the Zeonor surface where the amino dextran network acquires an outward orientation, favorable for an accessible covalent immobilization of the FK506-CO_2_H. The effect of the ADLC concentration in the patterning solution was examined over the 10–100 μg/mL range and tested by the response of the biosensor in the absence of FK506 (*B*_0_). As shown in Figure 3a, the best coating efficiency was obtained with a concentration of 50 μg/mL of ADLC, using a fixed incubation time of 30 min, while higher concentrations do not lead to increased absorption of conjugate on the surface.

### FK506-CO_2_H Immobilization

Immobilization of FK506-CO_2_H was carried out onto ADLC-Zeonor substrates after activation of the hapten with the classical carbodiimide reaction in 2-(*N*-morpholino)ethanesulfonic acid (MES) buffer. Figure 3b depicts the biosensor response in the absence of FK506 (*B*_0_) when the hapten is conjugated to the aminated BTL2-dextran using 0.1 M of EDC in the absence and in the presence of 50 mM of NHS. As it can be observed, the efficiency of the EDC-mediated reaction improves notably in the presence of NHS. This behavior can be attributed to the fact that the ester formed between FK506-CO_2_H and NHS is more stable against hydrolysis in aqueous media than the one involving EDC and FK506-CO_2_H.^31^

To find the optimal amount of hapten and EDC for coupling to ADLC-Zeonor substrates, different concentrations of FK506-CO_2_H (50, 100, and 200 μg/mL) and EDC (62.5, 100, and 200 μM), were investigated. The NHS concentration was fixed at 50 mM because the rate-determining step of the carboxylate activation mechanism is its nucleophilic addition to EDC. The FK506-ADLC-Zeonor waveguides were tested using the FLI assay, and the substrates coupled with 100 μg/mL FK506-CO_2_H and 100 μM EDC resulted in the highest and most sensitive signal responses (Figure 3c,d). This observation also suggests that the signal responses of FLI could be adjusted by controlling the amount of FK506-CO_2_H added to the coupling solution. The reproducibility of the coupling protocol was evaluated by performing FLI calibration sets using different batches of activated Zeonor slides using the same protocol. The results showed no statistical variance between different batches prepared by different researchers on different days (RSD < 15.8%).

### Selection of the Blocking Agent

**Figure 4:**
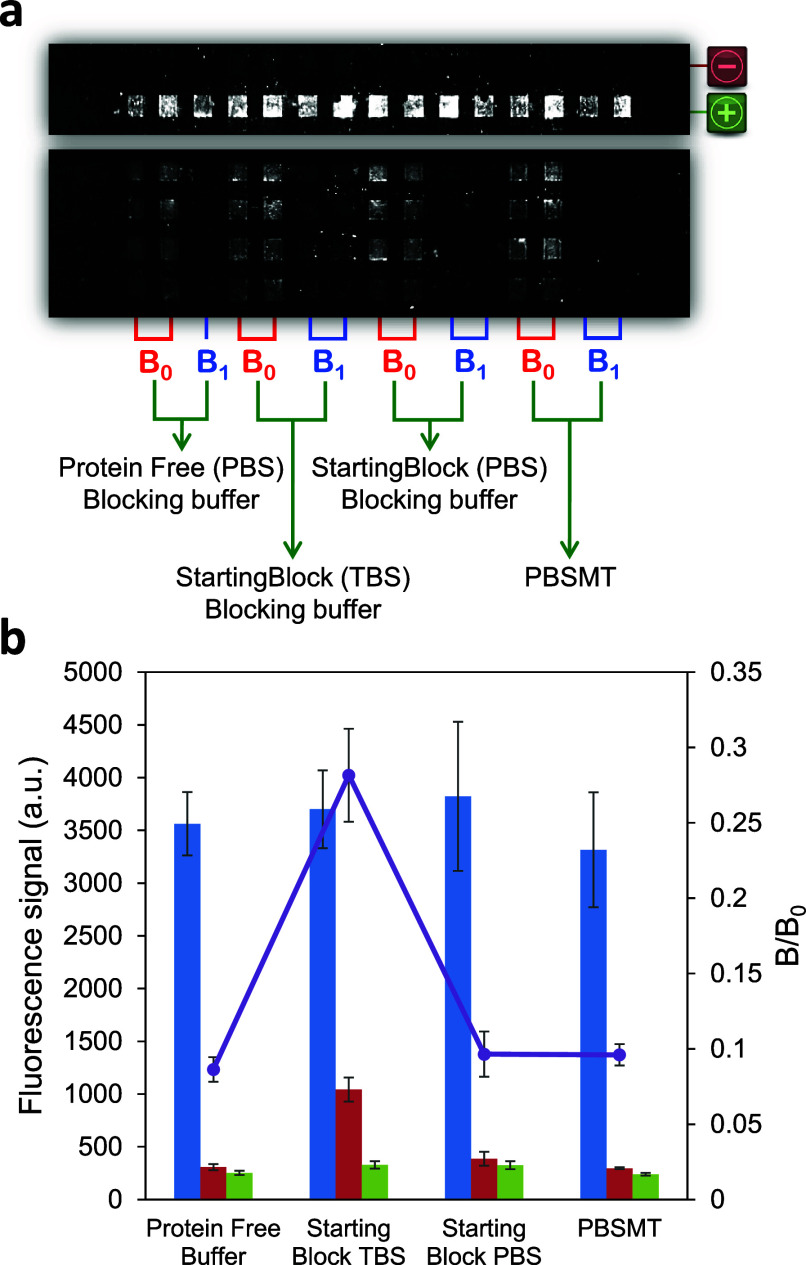
(a) Biochip image using different blocking buffers. Zeonor slide was patterned with 50 μg/mL of aminated dextran-BTL2 conjugate and 100 μg/mL of FK506-CO_2_H (immunoassay region), 30 μg/mL biotin (as positive control, green “+” symbol), or MES buffer (as negative control, red “–” symbol). *B*_0_ (0 ng/mL FK506) and *B*_1_ (100 ng/mL FK506) and using 2.5 μg/mL Alexa Fluor 647-anti-IgM as a revealing agent. (b) Comparison of the efficiency of the blocking buffer solutions tested on the *B*_1_/*B*_0_ ratio (open symbols). The blue bars correspond to the fluorescence signal in the absence of FK506 (*B*_0_), the red bars correspond to the signal in the presence of 100 ng/mL of FK506 (*B*), the green bars correspond to the signal of the background, and the purple circles correspond to the *B*/*B*_0_ ratio. Results are shown as mean ± standard error of the mean (*n* ≥ 4).

In order to minimize the nonspecific binding of bioreagents on the chip patterned surface, which could lead to false positives, four blocking agents were tested: (a) protein-free PBS blocking buffer (PBSPF), (b) StartingBlock TBS blocking buffer (TBSS), (c) StartingBlock PBS blocking buffer (PBSS), and (d) PBS with 0.05% T20 and 0.3% powdered nonfat milk (PBSTM). Overall, the four blocking agents prevent nonspecific binding during the assay. There is no significant difference in the fluorescence intensity obtained in the absence of analyte (*B*_0_) when they are included in each assay (Figure 4). However, blocking the Zeonor surface with PBSPF provided the best results in terms of reproducibility (RSD < 20%) and sensitivity (the lowest *B*/*B*_0_ ratio for a *B* = 100 ng/mL of FK506).

### Analytical Characteristics of Developed Biosensor

**Figure 5:**
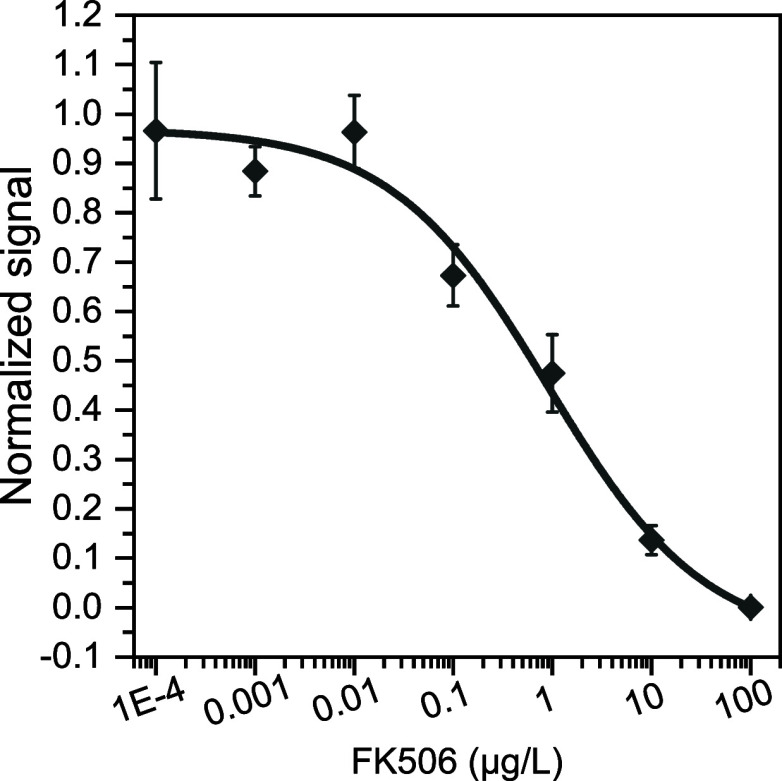
Competitive calibration curve. The assay was performed with FK506 (0.0001–100 μg/L) in the presence of 0.5 μg/mL of anti-FK506 and antibiotin antibodies. A mixture of 2.5 μg/mL Alexa Fluor 647-anti-IgM and Alexa Fluor 647-anti-IgG was used as the tracer solution. Results are shown as the mean ± standard error of the mean (*n* ≥ 3).

Figure 5 shows the competition inhibition curve obtained under the optimized conditions using FK506 standard solutions in the 0.0001–100 μg/L range. The obtained IC50 values and LOD values of the immunoassay were 0.9 and 0.02 ng/mL, respectively. The dynamic range, calculated from 20 to 80% inhibition, spanned from 0.07 to 11.3 ng/mL. Current clinical recommendation for FK506 measurements in transplanted patients involves a limit of quantification of 1 ng/mL to provide reliable amounts of the immunosuppressant drug during low-dose therapy.^32^ In this regard, the biosensor developed herein would be sensitive enough to monitor FK506 in real world samples (even with up to 14-fold dilution if required to avoid a matrix effect), matching or improving the LOD of previously described methods^33,34^ and opening the door to simplify the current surface modification protocols to manufacture functional POC devices based on COCs such as Zeonor (Table S1).

Assay selectivity was assessed with two ISDs typically coadministered with FK506 in transplanted patients (Figure S8). In both cases the two drugs exhibited negligible cross reactivities.

Additionally, the feasibility of biosensor regeneration was evaluated (Figure S9). The device can reliably detect FK506 over at least three cycles without significant loss of performance after treatment with a 50 mM NaOH solution at the end of each cycle, thereby confirming its suitability for semicontinuous operation in POC applications.

## Conclusions

This work demonstrates the applicability of ADLC conjugates as an efficient alternative for the functionalization of hydrophobic surfaces, such as COCs. The immobilization of custom-made amino-BTL2 conjugates onto planar plastic substrates is fast, straightforward, and reproducible. Further derivatization with FK506 yields a highly sensitive immunoassay for this drug. Therefore, the noncovalent immobilization of immunoreagents by way of their conjugates to a dextran-modified lipase represents an attractive alternative to conventional methods for heterogeneous immunoassays.

The novel aminodextran-lipase-coated COC substrates pave the way to develop on-a-chip immunosensors for point-of-care testing that meet their stringent requirements, as they have shown high reproducibility (mean interslide RSD of 16%), rapid substrate activation protocol (30 min), small volume (390 μL), and preservation of the optical properties of the COC substrate, among others. Furthermore, the method described herein represents a significant step toward the development of evanescent wave-based devices that can be integrated into multiplexed sensor systems.

## Supporting Information Available

Details about the COC surface modification by oxygen plasma, photochemical grafting, and amino dextran-lipase conjugate; details about the biosensor development; scheme of the FK506 immunoassay protocol; comparison of the hydrophobicity of Zeonor before and after the plasma treatment; biochip images for the analysis of FK506 using the developed immunoarray; ATR-FTIR results; comparative summary of different methods reported for COC surface activation (PDF)
