# Supplementary material for: Antibody-Based Array for Tacrolimus Immunosuppressant Monitoring with Planar Plastic Waveguides Activated with an Aminodextran-Lipase Conjugate
Source: Anal Chem. 2024 Aug 22;96(35):14142–9. doi: 10.1021/acs.analchem.4c02028 (PMC11375619; doi:10.1021/acs.analchem.4c02028)
Supplement: Supplementary file 1 — ac4c02028_si_001.pdf [file ac4c02028_si_001.pdf]

Supporting Information

Antibody-Based Array for Tacrolimus Immunosuppressant Monitoring with Planar Plastic Waveguides Activated with an Aminodextran-Lipase Conjugate

Bettina Glahn-Martínez,[1‡] Sonia Herranz,[1‡] Elena Benito-Peña,[1*] Guillermo Orellana,[2*] and Maria C. Moreno-Bondi[1†]

[1] Department of Analytical Chemistry, Faculty of Chemistry, Universidad Complutense de Madrid, Plaza de las Ciencias 2, 28040 Madrid, Spain

[2] Department of Organic Chemistry, Faculty of Chemistry, Universidad Complutense de Madrid, Plaza de las Ciencias 2, 28040 Madrid, Spain

[‡] Equally contributed

[†] The late

*** Correspondence:** elenabp@ucm.es, phone: +34-913-944-186 (E. Benito-Peña); gorellana@ucm.es, phone: +34-913-944-220 (G. Orellana)

Contents

1. Materials and Methods **2**

1.1. Reagents and Solutions **2**

1.2. Instrumentation **2**

1.3. COC surface modification **3**

1.3.1. Oxygen plasma treatment **3**

1.3.2. Photochemical grafting **3**

1.3.3. Surface amination using an amino dextran-lipase conjugate **3**

1.4. Biosensor development **3**

1.4.1. Antigen Immobilization onto the Zeonor Activated Sensing Surface **3**

1.4.2. Assay protocol **4**

Figure S1 .... **4**

Figure S2 .... **4**

Figure S3 .... **5**

Figure S4 .... **5**

Figure S5 .... **6**

Figure S6 .... **6**

Figure S7 .... **7**

Figure S8 .... **7**

Figure S9 .... **8**

Table S1 .... **9**

References **10**

1. Materials and Methods

1.1. Reagents and Solutions

Monoclonal mouse IgM antibody against FK506 was supplied by Santa Cruz Biotechnology (Paso Robles, CA, USA). The IgG fraction monoclonal mouse anti-biotin, Alexa Fluor 647-conjugated affinity-purified rabbit anti-mouse IgG, and Alexa Fluor 647-conjugated affinity-purified donkey anti-mouse IgM were from Jackson ImmunoResearch (West Grove, PA, USA). Tacrolimus (FK506) was from by Sinoway Industrial (Xiamen, Fujian, China). Benzophenone (BP), azobis(isobutyronitrile) (AIBN), and 2-aminoethyl methacrylate (2-AEM) were provided by Acros Organics (Geel, Belgium). Phosphate-buffered saline (PBS) (10 mM, pH 7.4), Tween-20 (T20), 1-ethyl-3-(3-(dimethylamino)propyl)carbodiimide (EDC), *N*-hydroxysuccinimide (NHS), 2-(*N*-morpholino)ethanesulfonic acid hydrate (MES), D-biotin, and 3-aminopropyltriethoxysilane (APTES) were from Sigma-Aldrich (St. Louis, MO, USA, now Merck). Pierce protein-free PBS blocking buffer (PBSPF), StartingBlock PBS blocking buffer (PBSS), and StartingBlock TBS blocking buffer (TBSS) were obtained from Thermo Fisher Scientific (Rockford, IL, USA). HPLC-grade methanol (MeOH) was supplied by Panreac Quimica SA (Barcelona, Spain).

BTL2 was expressed in *Escherichia coli*, produced and purified as described elsewhere.[1] Then, BTL2 was modified with amino dextran to obtain ADLC using a previously reported method.[2] Zeonor® microscope slides (dimensions 25 × 75 × 1 mm) were kindly donated by Microfluidic ChipShop (Germany).

Biotin and FK506 stock solutions were prepared in dimethylsulfoxide (DMSO) (1 mg/mL) and stored at −20 °C. FK506 standard solutions for calibration purposes were prepared daily upon diluting the stock solutions in PBS. Water was purified with a Milli-Q system (Millipore, Bedford, MA). All other chemicals used were of analytical reagent grade.

1.2. Instrumentation

The hydrophilicity of the surface was assessed by measuring the water contact angles at room temperature with a goniometer (Dataphysics OCA 15 Plus, Dataphysics Instruments GmbH, Germany). The contact angle between a 2 µL drop of deionized water and the substrate was measured immediately after the contact was made to minimize evaporation. Five drops of water were placed on the substrate, and the contact angle of each drop's left and right sides was optically measured (SCA 20 software, Dataphysics Instruments) and averaged.

Fourier Transform Infrared (FTIR) spectra of the polymers were obtained using a Perkin Elmer Spectrum 100 FTIR spectrometer equipped with a universal ATR sampling accessory (Perkin Elmer, UK). Spectra were recorded with a 4 cm^{-1} resolution in the 4000 to 400 cm^{-1} range.

Atomic force microscopy (AFM) was used to evaluate the coating of Zeonor surfaces with the ADLC. Substrates were characterized using a multimode AFM Multimode Nanoscope III A (Bruker) in tapping mode with Si_3N_4 tips (UCM Central Instrumentation Facilities). The size of the analyzed surfaces was 2 µm x 2 µm.

Fluorescence imaging was carried out using a Leopard Array Biosensor (Hanson Technologies, USA), a commercial version of the NRL Array Biosensor prototype.[3] The instrument is equipped with a 635 nm diode laser source (LAS-635-15, Lasermax). The excitation beam is focused on the edge of the microscope slide waveguide. The fluorescence array intensity developed on the slide is filtered using a long-pass (665 nm) and a band-pass filter (700 ± 35 nm) and measured using a CCD camera (Retiga 1300, Q-Imaging). The system is fully automated and uses two six-chamber reservoir modules for the samples and tracers, respectively. The slide is mounted vertically and pressed against a six-channel gasket molded in poly(dimethylsiloxane) (PDMS, NuSil Silicone Technology), forming six assay flow channels. Each channel is connected on one end to a peristaltic pump and on the other to a 2-way valve that switches between the sample and the tracer that flows through the channels.[3] The acquired image is analyzed using a proprietary software control interface (HLAB, Hanson Technologies) that controls the microfluidic system. The locations and intensities of the fluorescent spots allow for the identification and quantification of FK506 concentration in the samples.[4] A scheme of the array platform is shown in **Figure S2b**.

1.3. COC surface modification

This study tested cyclic olefin copolymer (COC) Zeonor® (a trademark of Zeon Corp., Tokio, Japan) slides to develop a suitable heterogeneous immunoassay for FK506 analysis. For this purpose, three procedures have been studied for activating the Zeonor slides' surfaces, considering the hydrophobic and unreactive nature of polyolefin materials.

1.3.1. Oxygen plasma activation and functionalization using 3-aminopropyltriethoxysilane

Oxidation of polymer surfaces by oxygen plasma treatment is a popular technique for the creation of functional groups which facilitate the immobilization of a variety of reagents, such as silanes,[5] that will fatherly attach carboxylated FK506 (FK506-CO_2H) [6] to the surface.

Oxygen plasma treatment of the Zeonor slides was performed by reactive-ion etching (RIE) using a PlasmaPro NGP80 Etch & Deposition Tool. The surface was oxidized with a 120 W oxygen plasma at 60 mTorr and 10 cm^3/min for 3–6 min. The activated slides were immersed in a solution containing 3% of APTES in MeOH–water (95:5, v/v) for 1 h at room temperature (RT) [7] (**Figure S1a**). The aminated slides were rinsed three times with clean MeOH and stored in the same solvent at 4 ºC until use.

1.3.2. Photochemical grafting

COC surfaces were modified using a sequential two-step photografting procedure previously described.[8,9] Briefly, in 200 µL-size wells placed onto thermoplastic substrates, photo-grafting at 312 nm (Spectroline 312A UV table) was initiated by either benzophenone (BP) or azobisisobutyronitrile (AIBN) in MeOH–water (90:10, v/v) at different concentrations (0.5–5%, w/v) and UV irradiation times (5–20 min). Subsequently, the wells were rinsed with MeOH to remove the excess initiator. Then, a 0.1 mol/L deoxygenated solution of 2-AEM in MeOH–water (90:10, v/v) was added to each activated well and re-illuminated with UV radiation (312 nm) for 30 min. Finally, the wells were extensively washed with water to remove the unreacted 2-AEM. The reaction scheme is depicted in **Figure S1b**.

1.3.3. Surface amination using an amino dextran-lipase conjugate

Described in the main text.

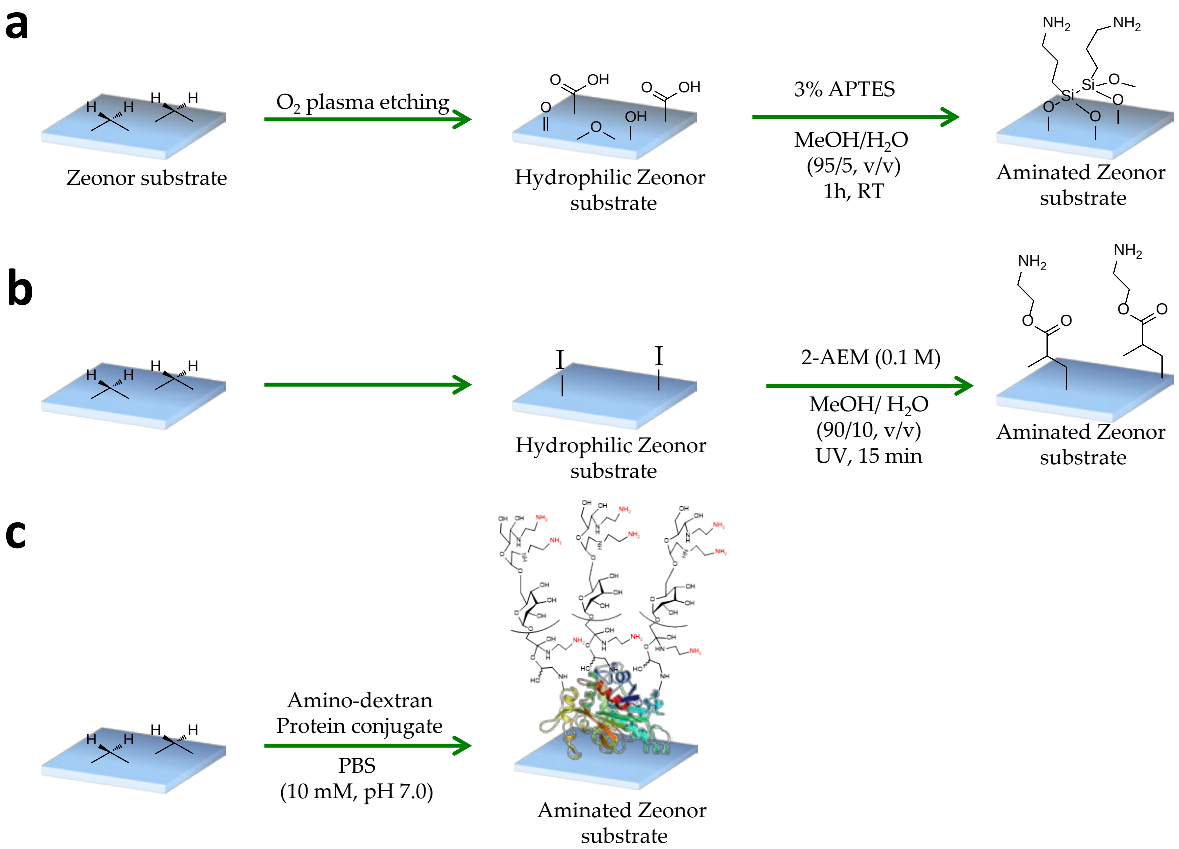

Figure S1 Zeonor surface modification using **(a)** an oxygen plasma followed by 3-aminopropyltriethoxysilane treatment; **(b)** photochemical grafting of 2-aminoethyl methacrylate, and **(c)** direct surface amination using an amino-dextran lipase conjugate.

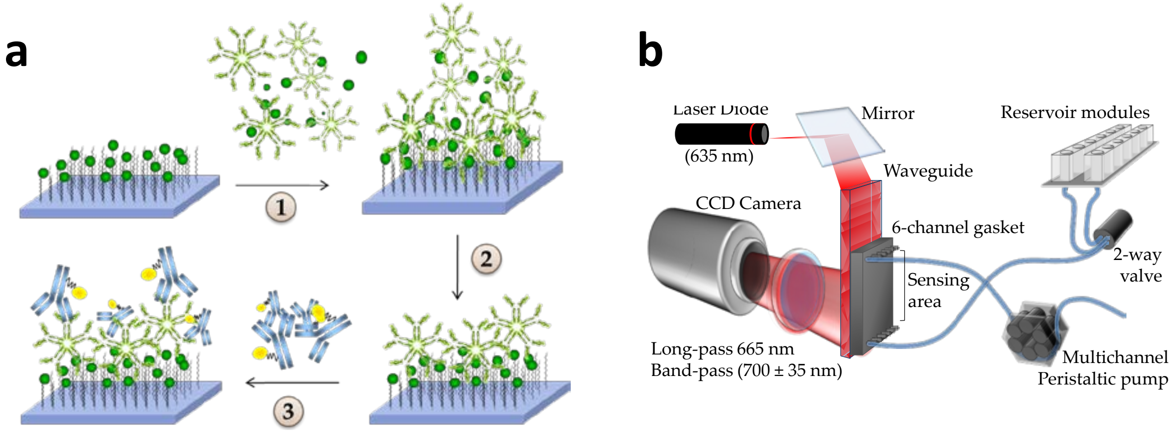

Figure S2 (a) Scheme of the tacrolimus immunoassay protocol: (1) the sample is incubated with the antibodies on the surface containing the bound hapten; (2) the excess of unbound bioreagents is washed away; (3) the labelled antibodies are injected and, after incubation, the excess is rinsed off and the image is acquired. **(b)** Scheme of the automated array biosensor platform used for the measurements. Adapted with permission from reference 12.

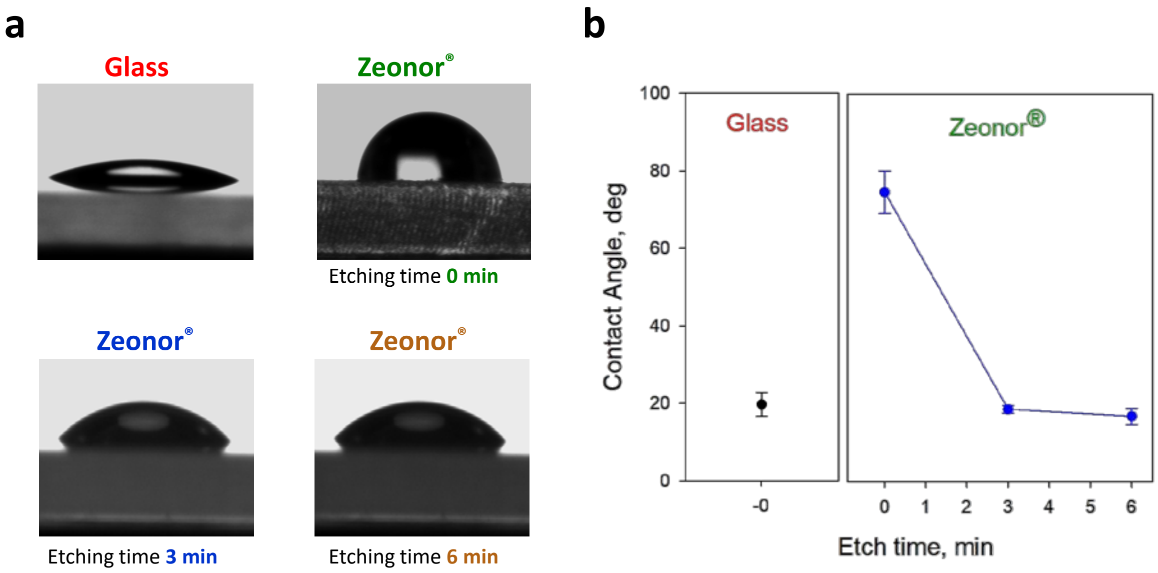

Figure S3 (a) Comparison of the hydrophobicity of Zeonor before and after the plasma treatment. **(b)** Effect of the treatment time on the contact angle of modified Zeonor surface.

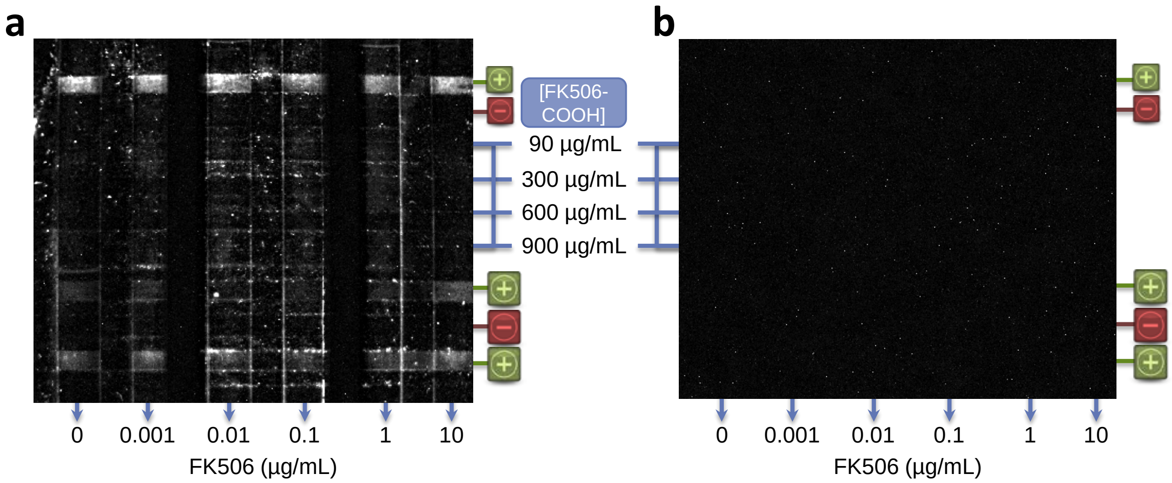

Figure S4 Biochip images for the analysis of FK506 using the developed immunoarray. The silanized Zeonor chip was patterned with FK506 (90–900 µg/mL), biotin (30 µg/mL) as positive control, and MES buffer as a negative control. The slide was assayed with different concentrations of FK506 (0.001–10 µg/L) in the presence of 0.4 µg/mL of anti-FK506 and 0.4 µg/mL of anti-biotin (positive control) antibodies. Tracer solution: 2.5 µg/mL AlexaFluor647-labeled anti-mouse IgG and anti-mouse IgM detection antibodies cocktail. **(a)** Zeonor with 3 min of plasma treatment. **(b)** Zeonor with 6 min of plasma treatment.

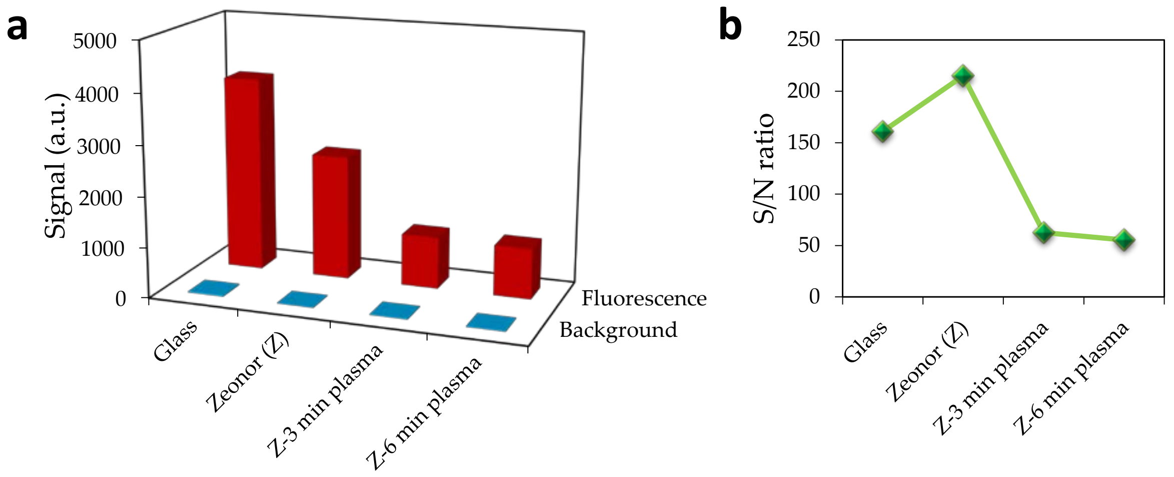

Figure S5 (a) Fluorescence intensity obtained for 0.5 µg/mL dilution of Alexa Fluor anti-IgG, using as waveguides: glass, original Zeonor (Z), Zeonor after plasma treatment for 3 min (Z-3), or Zeonor after 6 min plasma treatment (Z-6). **(b)** Signal-to-noise ratio (S/N) obtained using the different optical-waveguides: glass; untreated Zeonor (Z); Zeonor after plasma treatment for 3 min (Z-3); Zeonor after 6 min plasma treatment (Z-6). Background signal was measures at both sides of the channel.

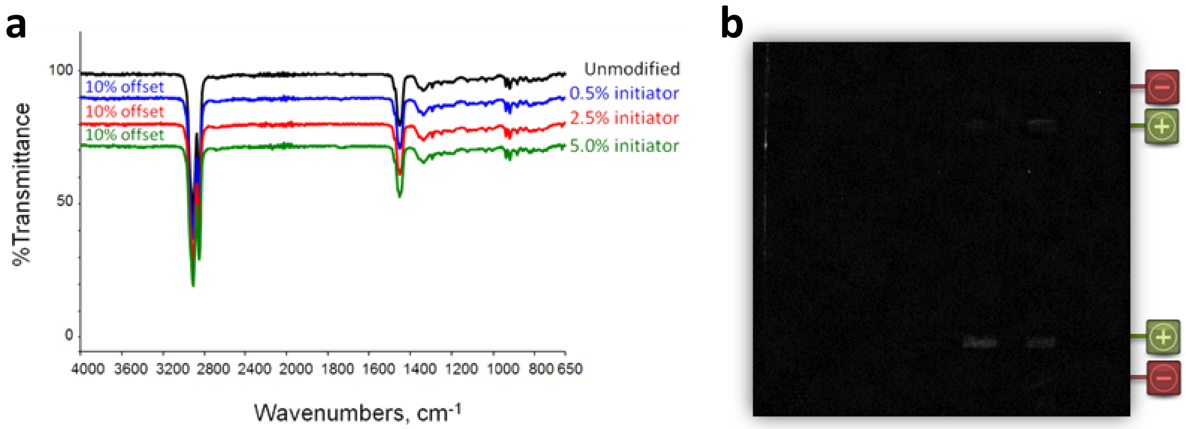

Figure S6 (a) ATR-FTIR of the original Zeonor (black line) and of the Zeonor treated with 0.5% benzophenone (BP; blue), 2.5% BP (red line), or 5.0% BP (green line) and 2-AEM (0.1 mol/L). **(b)** Image of the heterogeneous fluorescence immunoassay. The Zeonor surface was functionalized with FK506-CO_2H (90–900 µg/mL), biotin (30 µg/mL) as a positive control, or MES buffer as a negative control. The assay was performed with FK506 (0.001–100 µg/L) in the presence of 0.5 µg/mL anti-FK506 and anti-biotin. A mixture of 2.5 µg/mL AlexaFluor 647-anti-IgM and Alexa Fluor 647-anti-IgG was used as tracer solution.

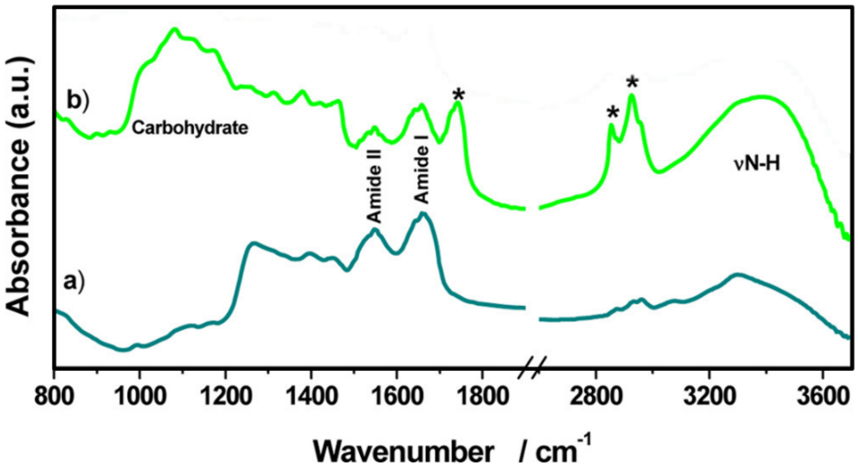

Figure S7 FT-IR spectra for **(a)** BTL2 and **(b)** aminated dextran–BTL2 conjugate. The asterisks indicate sucrose laurate bands. Adapted with permission from Herranz *et al.* Anal. Chem. 2013, 85, 15, 7060–7068. Copyright 2024 American Chemical Society.

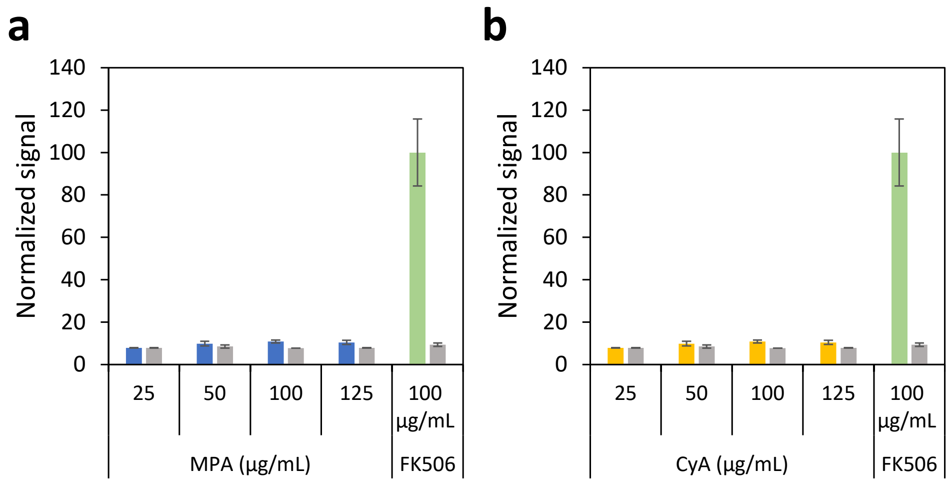

Figure S8 Cross reactivity with the immunosuppressants **(a)** mycophenolic acid (MPA) and **(b)** cyclosporin A (CyA) have been analyzed. The assay was performed after functionalizing the ADLC with different concentrations (25-125 µg/mL) of the immunosuppressants.

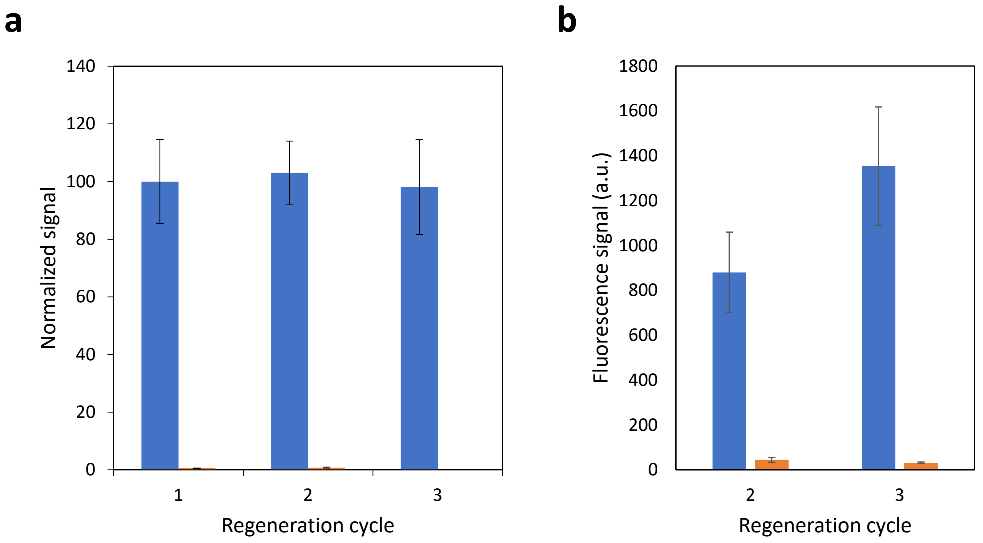

Figure S9 Evaluation of the regeneration of the assay surface after using 800 µL of **(a)** a 50 mM NaOH solution or **(b)** a 0.1 M Glycine/HCl (pH 2.2) solution. Blue bars correspond to the fluorescence signal after the assay and the orange bars after surface regeneration. In (a) the maximum fluorescence signals (B_0) are normalized against the first cycle.

Table S1. Comparison of different methods to activate COC surfaces described in the literature.

COC	Added groups	Method	Process duration	Further immobilization	Ref.
Zeonor	COOH/NH_2/Br	Photografting aryldiazonium salts	70 min	–	13
Topas	Siloxane	Photografting TMSPMA	>4 h	HSNs	14
Zeonor	NH_2 or PEG	PECVD + silane/PEG	5 min	–	15
Zeonor Zeonex	NH_2	Oxygen plasma + silane	80–105 min	Antibodies	16
Zeonor COP	Epoxy	Oxygen plasma + silane	>16 h	BSA or streptavidin	17
Zeonor Zeonex	COOH, C=O, OH	Ozone oxidation	1 h	DNA	18
Zeonor	Azide or alkyne	Ozone oxidation or oxygen plasma + carbodiimide reaction	> 16 h	TAMRA	19
Topas	Hydroperoxide, carbonyl, carboxylic acid, peroxyacid	Low-pressure microwave plasma	6–11 min	Metals (Al and Cu)	20
Zeonex	NH_2	KOH + silane	95 min	Antibodies	21
Topas		Dynamic HEC coating	5 min	–	22
Zeonor	NH_2	i) Plasma + silane<br>ii) Photochemical grafting<br>**iii) Amino dextran-lipase conjugate**	i) 70 min<br>ii) 60 min<br>**iii) 30 min**	FK506-COOH	**This work**

Key: HEC: hydroxyethyl cellulose; HSNs: hollow silica nanoparticles; PECVD: plasma enhanced chemical vapor deposition; PEG: polyethylene glycol; TAMRA: tetramethylrhodamine; TMSPMA: 3-(trimethoxysilyl)propyl methacrylate.

References

(1) Schmidt-Dannert, C.; Rúa, M. L.; Atomi, H.; Schmid, R. D. Thermoalkalophilic Lipase of Bacillus Thermocatenulatus. I. Molecular Cloning, Nucleotide Sequence, Purification and Some Properties. *Biochim. Biophys. Acta* **1996**, *1301* (1), 105–114. https://doi.org/10.1016/0005-2760(96)00027-6.

(2) Herranz, S.; Marciello, M.; Olea, D.; Hernández, M.; Domingo, C.; Vélez, M.; Gheber, L. A.; Guisán, J. M.; Moreno-Bondi, M. C. Dextran–Lipase Conjugates as Tools for Low Molecular Weight Ligand Immobilization in Microarray Development. *Anal. Chem.* **2013**, *85* (15), 7060–7068. https://doi.org/10.1021/ac400631t.

(3) Ligler, F. S.; Sapsford, K. E.; Golden, J. P.; Shriver-Lake, L. C.; Taitt, C. R.; Dyer, M. A.; Barone, S.; Myatt, C. J. The Array Biosensor: Portable, Automated Systems. *Anal. Sci.* **2007**, *23* (1), 5–10. https://doi.org/10.2116/analsci.23.5.

(4) Taitt, C. R.; Malanoski, A. P.; Lin, B.; Stenger, D. A.; Ligler, F. S.; Kusterbeck, A. W.; Anderson, G. P.; Harmon, S. E.; Shriver-Lake, L. C.; Pollack, S. K.; Lennon, D. M.; Lobo-Menendez, F.; Wang, Z.; Schnur, J. M. Discrimination between Biothreat Agents and ‘near Neighbor’ Species Using a Resequencing Array. *FEMS Immunol. Med. Microbiol.* **2008**, *54* (3), 356–364. https://doi.org/10.1111/j.1574-695X.2008.00486.x.

(5) Gandhiraman, R. P.; Gubala, V.; O’Mahony, C. C.; Cummins, Th.; Raj, J.; Eltayeb, A.; Doyle, C.; James, B.; Daniels, S.; Williams, D. E. PECVD Coatings for Functionalization of Point-of-Care Biosensor Surfaces. *Vacuum* **2012**, *86* (5), 547–555. https://doi.org/10.1016/j.vacuum.2011.08.014.

(6) Salis, F.; Descalzo, A. B.; Benito-Peña, E.; Moreno-Bondi, M. C.; Orellana, G. Highly Fluorescent Magnetic Nanobeads with a Remarkable Stokes Shift as Labels for Enhanced Detection in Immunoassays. *Small* **2018**, *14* (20), 1703810. https://doi.org/10.1002/smll.201703810.

(7) Manning, M.; Redmond, G. Formation and Characterization of DNA Microarrays at Silicon Nitride Substrates. *Langmuir* **2005**, *21* (1), 395–402. https://doi.org/10.1021/la0480033.

(8) Ma, H.; Davis, R. H.; Bowman, C. N. A Novel Sequential Photoinduced Living Graft Polymerization. *Macromolecules* **2000**, *33* (2), 331–335. https://doi.org/10.1021/ma990821s.

(9) Stachowiak, T. B.; Svec, F.; Fréchet, J. M. J. Patternable Protein Resistant Surfaces for Multifunctional Microfluidic Devices via Surface Hydrophilization of Porous Polymer Monoliths Using Photografting. *Chem. Mater.* **2006**, *18* (25), 5950–5957. https://doi.org/10.1021/cm0617034.

(10) Hermanson, G. T. The Chemistry of Reactive Groups. In *Bioconjugate Techniques (Second Edition)*; Hermanson, G. T., Ed.; Academic Press: New York, 2008; pp 169–212. https://doi.org/10.1016/B978-0-12-370501-3.00002-3.

(11) Herranz, S.; Marazuela, M. D.; Moreno-Bondi, M. C. Automated Portable Array Biosensor for Multisample Microcystin Analysis in Freshwater Samples. *Biosens. Bioelectron.* **2012**, *33* (1), 50–55. https://doi.org/10.1016/j.bios.2011.12.016.

(12) Herranz de Andrés, S. *Doctoral Thesis. “Nuevas Herramientas Analíticas Para La Determinación de Contaminantes En El Medio Ambiente y En Alimentos”;* Universidad Complutense de Madrid, 2013.

(13) Brisset, F.; Vieillard, J.; Berton, B.; Morin-Grognet, S.; Duclairoir-Poc, C.; Le Derf, F. Surface Functionalization of Cyclic Olefin Copolymer with Aryldiazonium Salts: A Covalent Grafting Method. *Applied Surface Science* **2015**, *329*, 337–346. https://doi.org/10.1016/j.apsusc.2014.12.060.

(14) Yan, J.; Zhao, C.; Ma, Y.; Yang, W. Covalently Attaching Hollow Silica Nanoparticles on a COC Surface for the Fabrication of a Three-Dimensional Protein Microarray. *Biomacromolecules* **2022**, *23* (6), 2614–2623. https://doi.org/10.1021/acs.biomac.2c00354.

(15) Gubala, V.; Siegrist, J.; Monaghan, R.; O’Reilly, B.; Gandhiraman, R. P.; Daniels, S.; Williams, D. E.; Ducrée, J. Simple Approach to Study Biomolecule Adsorption in Polymeric Microfluidic Channels. *Anal. Chim. Acta* **2013**, *760*, 75–82. https://doi.org/10.1016/j.aca.2012.11.030.

(16) Raj, J.; Herzog, G.; Manning, M.; Volcke, C.; MacCraith, B. D.; Ballantyne, S.; Thompson, M.; Arrigan, D. W. M. Surface Immobilisation of Antibody on Cyclic Olefin Copolymer for Sandwich Immunoassay. *Biosens. Bioelectron.* **2009**, *24* (8), 2654–2658. https://doi.org/10.1016/j.bios.2009.01.026.

(17) Hager, R.; Forsich, C.; Duchoslav, J.; Burgstaller, C.; Stifter, D.; Weghuber, J.; Lanzerstorfer, P. Microcontact Printing of Biomolecules on Various Polymeric Substrates: Limitations and Applicability for Fluorescence Microscopy and Subcellular Micropatterning Assays. *ACS Appl. Polym. Mater.* **2022**, *4* (10), 6887–6896. https://doi.org/10.1021/acsapm.2c00834.

(18) Diaz-Quijada, G. A.; Peytavi, R.; Nantel, A.; Roy, E.; Bergeron, M. G.; Dumoulin, M. M.; Veres, T. Surface Modification of Thermoplastics—towards the Plastic Biochip for High Throughput Screening Devices. *Lab Chip* **2007**, *7* (7), 856–862. https://doi.org/10.1039/B700322F.

(19) Faragher, R. J.; McKay, C. S.; Hoa, X. D.; Prikrylova, B.; Lopinski, G. P.; Figeys, D.; Veres, T.; Pezacki, J. P. Functionalizing Poly(Cyclic Olefins) Using Copper-Catalyzed Azide–Alkyne “Click” Reactions. *Can. J. Chem.* **2011**, *89* (5), 608–615. https://doi.org/10.1139/v11-015.
(20) Nikolova, D.; Dayss, E.; Leps, G.; Wutzler, A. Surface Modification of Cycloolefinic Copolymers for Optimization of the Adhesion to Metals. *Surf. Interface Anal.* **2004**, *36* (8), 689–693. https://doi.org/10.1002/sia.1737.
(21) Dixit, C. K.; Vashist, S. K.; O’Neill, F. T.; O’Reilly, B.; MacCraith, B. D.; O’Kennedy, R. Development of a High Sensitivity Rapid Sandwich ELISA Procedure and Its Comparison with the Conventional Approach. *Anal. Chem.* **2010**, *82* (16), 7049–7052. https://doi.org/10.1021/ac101339q.
(22) Zhang, J.; Das, C.; Fan, Z. H. Dynamic Coating for Protein Separation in Cyclic Olefin Copolymer Microfluidic Devices. *Microfluid. Nanofluidics* **2008**, *5* (3), 327–335. https://doi.org/10.1007/s10404-007-0253-5.
